# Identification of Pyroptosis-Related Genes and Immune Landscape in Myocardial Ischemia–Reperfusion Injury

**DOI:** 10.3390/biomedicines13092114

**Published:** 2025-08-29

**Authors:** Yanfang Zhu, Haoyan Zhu, Jia Zhou, Jiahe Wu, Xiaorong Hu, Chenze Li, Huanhuan Cai, Zhibing Lu

**Affiliations:** 1 Department of Cardiology, Zhongnan Hospital of Wuhan University, Wuhan 430071, China; zn001625@whu.edu.cn (Y.Z.); 2021203030006@whu.edu.cn (H.Z.); zhoujia@whu.edu.cn (J.Z.); 2015302180246@whu.edu.cn (J.W.); huxrzn@whu.edu.cn (X.H.); lichenze@whu.edu.cn (C.L.); 2 Institute of Myocardial Injury and Repair, Wuhan University, Wuhan 430071, China

**Keywords:** myocardial ischemia–reperfusion injury, pyroptosis, bioinformatic analysis, immune infiltration, molecular docking

## Abstract

**Background**: Cardiomyocyte death is a key factor in myocardial ischemia–reperfusion injury (MI/RI), and the expression patterns and molecular mechanisms of pyroptosis-related genes (PRGs) in ischemia–reperfusion injury are poorly understood. **Methods**: The mouse MI/RI injury-related datasets GSE61592 and GSE160516 were obtained from the Gene Expression Omnibus database, and differential expression analysis was performed on each to identify differentially expressed genes (DEGs). The DEGs were intersected with the PRGs obtained from GeneCards to identify differentially expressed PRGs in MI/RI. Enrichment analysis identified key pathways, while PPI network analysis revealed hub genes. The expression patterns and immune cell infiltration of hub genes were also investigated. The molecular docking prediction of key genes was performed using MOE software in conjunction with the ZINC small molecular compounds database. Key gene expression was validated in an external dataset (GSE4105), a mouse MI/RI model, and an HL-1 cell hypoxia/reoxygenation model via RT-qPCR. **Results**: A total of 29 differentially expressed PRGs were identified, which are primarily associated with pathways such as “immune system process”, “response to stress”, “identical protein binding”, and “extracellular region”. Seven key genes (*Fkbp10*, *Apoe*, *Col1a2*, *Ppic*, *Tlr2*, *Fstl1*, *Serpinh1*) were screened, all strongly correlated with immune infiltration. Seven FDA-approved small molecule compounds exhibiting the highest docking potential with each key gene were selected based on a comprehensive evaluation of S-scores and hydrogen bond binding energies. *Apoe*, *Tlr2*, and *Serpinh1* were successfully validated across external datasets, the mouse MI/RI model, and the cardiomyocyte H/R model. **Conclusions**: *Apoe*, *Tlr2*, and *Serpinh1* may be key genes involved in MI/RI-related pyroptosis. Targeting these genes may provide new insights into the treatment of MI/RI.

## 1. Introduction

Myocardial infarction remains a major global health challenge, with early reperfusion therapy being the most effective strategy to minimize infarct size [1]. However, reperfusion of the acutely ischemic myocardium can paradoxically trigger cardiomyocyte death, leading to arrhythmias, myocardial stunning, and microcirculatory obstruction and ultimately resulting in cardiac remodeling and impaired cardiac function [2]. This damage is commonly known as myocardial ischemia–reperfusion injury (MI/RI). The mechanisms underlying MI/RI are complex, with established contributing factors including oxidative stress [3], intracellular calcium overload [4], and rapid pH recovery [5]. Unfortunately, targeted therapies against these factors have not yielded satisfactory results, and there are currently no effective drugs available to prevent MI/RI [6].

The loss of cardiomyocytes is the fundamental factor causing irreversible cardiac damage in MI/RI. Regulated cardiomyocyte death (RCD) is the primary form of cell loss in MI/RI. Studies have shown that apoptosis [7], ferroptosis [8], PANoptosis [9], and pyroptosis [10] are involved in the disease progression of MI/RI. Pyroptosis is a form of inflammatory programmed cell death marked by gasdermin-mediated pore formation in the plasma membrane, resulting in cell swelling and death. This process also generates and releases inflammatory factors such as IL-1β and IL-18, triggering an inflammatory response [11]. Pyroptosis occurs in the early stages of reperfusion, triggering acute MI/RI. Studies have shown that the NLRP3 inflammasome in the canonical pyroptosis pathway can be activated as early as 20 min after reperfusion [12], and inhibiting NLRP3 one hour after reperfusion can significantly reduce infarct size [13]. Additionally, during MI/RI, inflammatory factors such as IL-1β and IL-18 are primarily released by non-cardiomyocytes [14], such as endothelial cells and leukocytes [15], thereby contributing to the establishment of the inflammatory state. These studies suggest that pyroptosis plays a crucial role in MI/RI, and targeting pyroptosis-related genes (PRGs) may be a potential strategy for the treatment of MI/RI. Therefore, it is necessary to identify PRGs in MI/RI, investigate their expression patterns and involvement in pathways, and search for potential therapeutic targets.

This study utilized transcriptomic analysis to identify differentially expressed PRGs in MI/RI-related datasets and explored the biological processes involving these genes through enrichment analysis. The relationship between key PRGs, immune cells, and immune responses was investigated through immune infiltration analysis. The key genes were subjected to molecular docking with the ZINC database using MOE software (version: 2022.02) to identify small molecular compounds with potential targeting effects. The expression of these genes was validated using external datasets, an animal MI/RI model, and a cellular hypoxia–reoxygenation (H/R) model. The purpose of this study is to explore the molecular biological mechanisms by which pyroptosis is involved in MI/RI and to provide new insights into potential therapeutic targets for MI/RI.

## 2. Materials and Methods

### 2.1. Data Source

The MI/RI-related datasets GSE61592 and GSE160516 (both from mouse models), as well as GSE4105 (from a rat model), were retrieved from the Gene Expression Omnibus (GEO) database (https://www.ncbi.nlm.nih.gov/; accessed on 10 June 2024). These datasets were selected based on the following criteria: (1) the experimental groups received MI/RI treatment; (2) the samples were derived from cardiac tissue; and (3) complete raw mRNA data were available. GSE61592 is based on the GPL6887 platform (Illumina MouseWG-6 v2.0 expression beadchip). GSE160516 is based on GPL23038 ([Clariom_S_Mouse] Affymetrix Clariom S Assay, Mouse (Includes Pico Assay)). GSE4105 is based on the GPL341 platform ([RAE230A] Affymetrix Rat Expression 230A Array). GSE61592 contains 3 control samples and 3 experimental samples. In the experimental group, myocardial ischemia was induced by ligating the left anterior descending (LAD) coronary artery for 90 min, followed by a reperfusion period of 72 h. GSE160516 contains 4 control samples and 4 experimental samples. GSE4105 contains 3 control samples and 3 experimental samples. In the experimental group, rats underwent ligation of the left anterior descending coronary artery (LAD) for 30 min, followed by reperfusion for 7 days. In this study, GSE61592 and GSE160516 were utilized for primary analysis, while GSE4105 served as an independent validation dataset.

### 2.2. Identification of DEGs and Differential Expression PRGs

Differentially expressed genes (DEGs) between the control and MI/RI groups were identified using the “Limma” package in R software (version: 4.3.2), based on an analysis of the MI/RI-related datasets. *p*-values were adjusted using the Benjamini–Hochberg method for multiple testing correction. The criteria for selecting DEGs were set as adjusted *p*-value < 0.05 and |Log2(FoldChange)| > 1. Genes related to pyroptosis with a relevance score > 1 in the GeneCards database were selected as PRGs. The differentially expressed PRGs were identified by intersecting the DEGs from the two datasets with the PRGs using an online Venn diagram tool (http://bioinformatics.psb.ugent.be/webtools/Venn/; accessed on 13 June 2024).

### 2.3. Functional and Pathway Enrichment Analysis

Gene Ontology [16] (GO) and Kyoto Encyclopedia of Genes and Genomes [17] (KEGG) enrichment analyses were performed using the “clusterProfiler” package in R. Pathways with *p* < 0.05 were considered significantly enriched.

### 2.4. Protein–Protein Interaction (PPI) Analysis and Identification of Key PRGs

The online database STRING [18] (accessed on 15 August 2024) was used for the PPI analysis of the differentially expressed PRGs, and Cytoscape software (version: 3.9.1) was employed to visualize the PPI network. The “Cytohubba” plugin in Cytoscape software was used to identify key PRGs. Four algorithms within the “Cytohubba” plugin (MCC, DMNC, MNC, and Degree) were applied for screening. The intersection of the results from these four algorithms was taken to determine the key PRGs.

### 2.5. Expression Pattern of Key PRGs in MI/RI

Box plots were used to display the differential expression trends in key PRGs in the GSE61592 and GSE160516 datasets. Pearson correlation analysis was conducted to examine the correlation of key PRGs in the GSE61592 and GSE160516 datasets, and the correlation coefficients were displayed in the form of heatmaps.

### 2.6. Identification of Immune Infiltration Landscape in MI/RI

The immune characteristic of key PRGs, including immune cell infiltration and immune response activity, in the GSE61592 and GSE160516 datasets was analyzed by single-sample gene set enrichment analysis (ssGSEA). A total of 23 immune cell infiltration signatures and 17 immune response activity signatures were obtained from the Immunology Database and Analysis Portal (ImmPort) (https://www.immport.org/home; accessed on 13 June 2024).

### 2.7. Molecular Docking of Small Molecular Compounds Targeting Key Genes

To screen for potential drugs targeting key genes, Molecular Operating Environment (MOE) software (version 2022.02) was used for molecular docking analysis and result visualization. FDA-approved drugs from the ZINC small molecule database (https://zinc.docking.org/catalogs/fda/; accessed on 15 April 2025) were used for docking simulations with the key genes. The 3D molecular structures of the key genes were either obtained from the RCSB PDB database (https://www.rcsb.org/; accessed on 15 April 2025) or predicted by AlphaFold (https://alphafold.ebi.ac.uk; accessed on 15 April 2025). Protein structures were prepared in MOE software by removing water molecules and adding hydrogen atoms. In MOE software, the Triangle Matcher method was used for molecular docking, with two parameters, London dG (quantifying van der Waals forces and hydrophobic interactions between ligand and receptor) and GBVI/WSA dG (calculating solvation free energy), employed to evaluate the binding affinity between the ligand and receptor. The London dG parameter was set to 10, while the GBVI/WSA dG parameter was set to 1. The S score is a comprehensive scoring function for predicting receptor–ligand binding free energy, based on the integration of the London dG and GBVI/WSA dG energy terms. In the docking results, the conformation with the highest S score and a hydrogen bond binding energy between the ligand and receptor > −5 kcal/mol was considered the optimal conformation.

### 2.8. Further Validation of Differentially Expressed PRGs in External Dataset

The GSE4105 dataset contained transcriptomic data of heart muscle tissue from rats that underwent MI/RI treatment and sham rats. The comparison between the experimental group and the control group was performed by an independent sample T-test. *p* < 0.05 was considered statistically significant.

### 2.9. Construction of MI/RI Mouse Model

All animal experiments in this study were approved by the Experimental Animal Ethics Committee of Zhongnan Hospital of Wuhan University (Ethics approval number: ZN2023201, approval date 30 October 2023). This study was conducted in strict accordance with the ARRIVE 2.0 guidelines for the reporting of animal research. Male C57BL/6 mice (*n* = 6, 8 weeks old) were purchased from Beijing Vital River Laboratory Animal Technology Co., Ltd. (Beijing, China) and randomly divided into sham and MI/RI groups (3 mice per group). The MI/RI mouse model was established by ligating the LAD for 30 min, followed by reperfusion for 24 h.

### 2.10. Cardiomyocyte Cell Line Culture and Hypoxia–Reoxygenation (H/R) Treatment

Mouse cardiomyocyte cell line HL-1 was purchased from BeNa Culture Collection. Cells were cultured in complete medium consisting of DMEM (Gibco, Invitrogen, Carlsbad, CA, USA), 10% fetal bovine serum (FBS, Gibco, Newcastle, Australia), and 1% penicillin–streptomycin (Sigma-Aldrich, St. Louis, MO, USA). After the cells grew to an appropriate density, they were cultured in glucose-free, serum-free medium and transferred to a three-gas incubator for 12 h of hypoxia. Afterward, the medium was replaced with complete medium, and cells were reoxygenated for 4 h.

### 2.11. Real-Time Quantitative Polymerase Chain Reaction

The expression of key PRGs in animal and cell models was detected using real-time quantitative polymerase chain reaction (qRT-PCR). Total RNA from cardiac tissue in animal models was extracted using Trizol (Thermo Fisher, Waltham, MA, USA). In cell models, total RNA was extracted using the FastPure Cell/Tissue Total RNA Isolation Kit V2 (Vazyme, Nanjing, China). Subsequently, reverse transcription was performed using the Hifair III 1st Strand cDNA Synthesis SuperMix for qRT-PCR (Ye Sen, China). qRT-PCR was performed using Hieff UNICON Universal Blue qPCR SYBR Green Master Mix (Ye Sen, China) for the fluorescent staining of cDNA. Gene expression levels were normalized to β-actin, and the relative fold changes were quantified via the 2^−ΔΔCt^ method. Details of all primers are presented in Supplementary File S1.

### 2.12. Statistical Analysis

All data are expressed as the mean ± standard error of the mean (SEM) based on a minimum of three independent experiments. Comparisons between two unpaired groups were performed using an independent sample t-test. *p* < 0.05 was considered statistically significant.

## 3. Results

### 3.1. Identification and Functional Enrichment of DEGs

**Figure 1:**
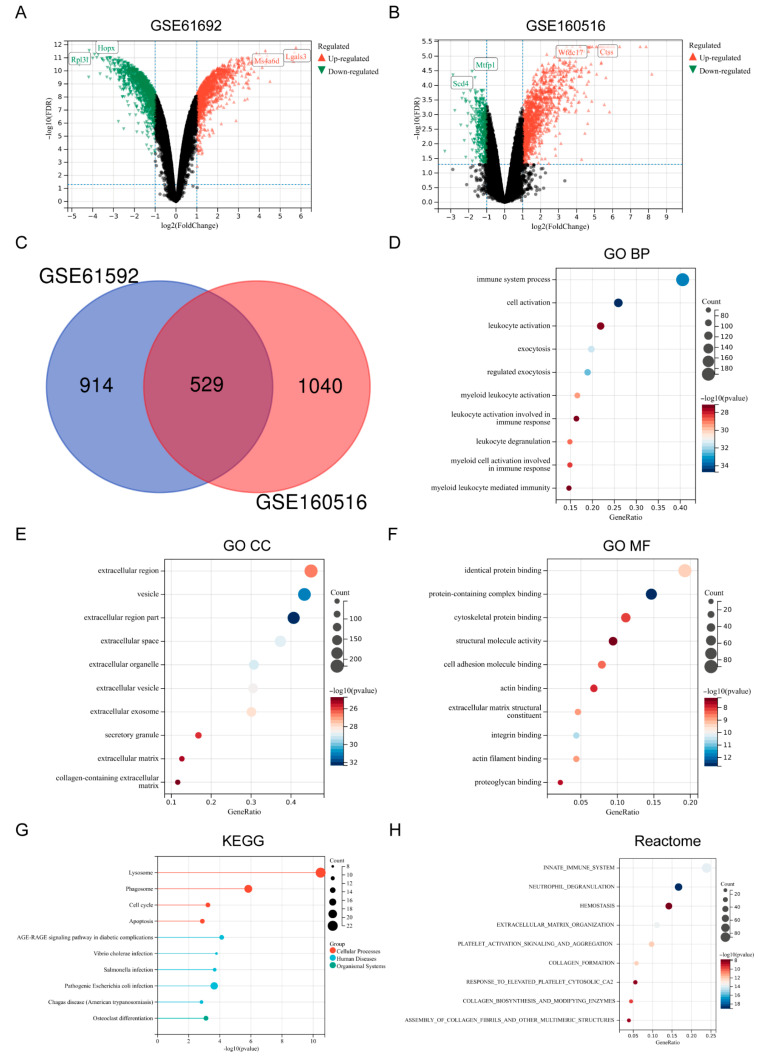
Identification and functional enrichment of DEGs. (**A**) Volcano plot of GSE61692. (**B**) Volcano plot of GSE160516. (**C**) Venn plot of key genes. (**D**) Bubble plot of DEGs in terms of GO BP (TOP10). (**E**) Bubble plot of DEGs in terms of GO CC (TOP10) (**F**) Bubble plot of DEGs in terms of GO MF (TOP10). (**G**) Bubble plot of DEGs in terms of KEGG (TOP10). (**H**) Bubble plot of DEGs in terms of Reactome (TOP10).

Based on the screening criteria of adjusted *p*-value < 0.05 and |Log2(FoldChange)| > 1, a total of 1443 DEGs were identified from the MI/RI-related dataset GSE61592, including 868 up-regulated genes and 575 down-regulated genes (Figure 1A). A total of 1580 DEGs were identified from the dataset GSE160516, including 1248 up-regulated genes and 332 down-regulated genes (Figure 1B). By taking the intersection of the DEGs from the two datasets, 529 key genes were identified (Figure 1C). GO Biological Process (BP) revealed that the key genes were primarily enriched in the “immune system process”, “cell activation”, and “leukocyte activation” pathways (Figure 1D). GO Cellular Component (CC) revealed that the key genes were primarily enriched in “extracellular region”, “vesicle”, and “extracellular region part” (Figure 1E). GO Molecular Function (MF) revealed that the key genes were primarily enriched in “identical protein binding”, “protein-containing complex binding”, and “cytoskeletal protein binding” (Figure 1F). KEGG enrichment analysis revealed that the key genes were primarily enriched in “Lysosome”, “Phagosome”, and “Apoptosis” (Figure 1G). Reactome enrichment analysis showed that the key genes were primarily enriched in “Innate immune system”, “Neutrophil degranulation”, and “Hemostasis” (Figure 1H). Detailed information about the key genes is presented in Supplementary File S2.

### 3.2. Identification and Functional Enrichment of Differential Expression PRGs

**Figure 2:**
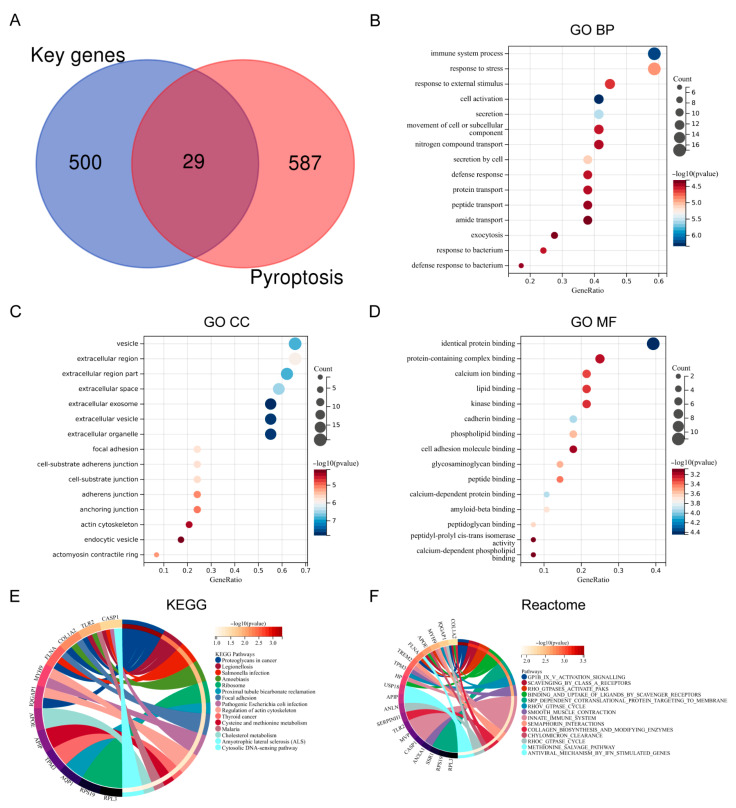
Identification and functional enrichment of differential expression PRGs. (**A**) Venn plot of differential expression PRGs. (**B**) Bubble plot of differential expression PRGs in terms of GO BP (TOP15). (**C**) Bubble plot of differential expression PRGs in terms of GO CC (TOP15). (**D**) Bubble plot of differential expression PRGs in terms of GO MF (TOP15). (**E**) Circle plot of KEGG pathway enrichment analysis of differential expression PRGs (TOP15). (**F**) Circle plot of reactome enrichment analysis of differential expression PRGs (TOP15).

The intersection of key genes with PRGs obtained from GeneCards identified 29 differentially expressed PRGs in the MI/RI-related datasets (Figure 2A). GO BP annotation revealed that the 29 differentially expressed PRGs were primarily enriched in “immune system process”, “response to stress”, and “response to external stimulus” (Figure 2B). GO CC annotation revealed that they were mainly enriched in “vesicle”, “extracellular region”, and “extracellular region part” (Figure 2C). GO MF annotation revealed that they were mainly enriched in “identical protein binding”, “protein-containing complex binding”, and “calcium ion binding” (Figure 2D). KEGG enrichment analysis revealed that the genes were primarily enriched in “Proteoglycans in cancer”, “Legionellosis”, and “Salmonella infection” (Figure 2E). Reactome enrichment analysis revealed that the genes were primarily enriched in “GP1B 1X V activation signalling”, “Scavenging by class a receptors”, and “Rho GTPases activate paks” (Figure 2F). Detailed information about the differential expression PRGs is presented in Supplementary File S3.

### 3.3. Construction of PPI Network and Identification of Key PRGs

**Figure 3:**
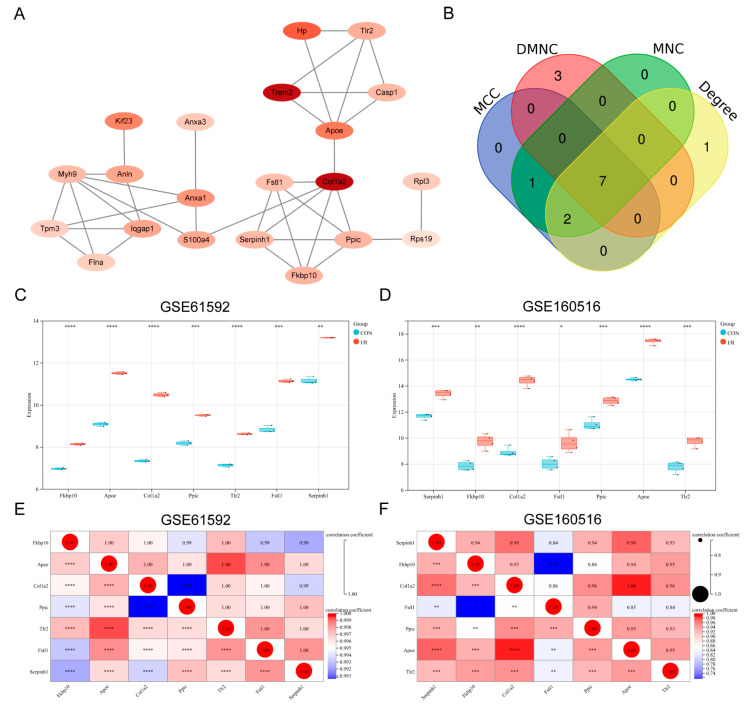
Construction of PPI network and identification of key PRGs. (**A**) PPI network of differential expression PRGs. (**B**) Venn plot of key PRGs. (**C**) Box plot of gene expression of key PRGs in GSE61592. (**D**) Box plot of gene expression of key PRGs in GSE160516. (**E**) Correlation heatmap of key PRGs in GSE61592. (**F**) Correlation heatmap of key PRGs in GSE160516. * *p* < 0.05; ** *p* < 0.01; *** *p* < 0.001; **** *p* < 0.0001.

Differentially expressed PRGs were analyzed for PPI using the STRING database, and the resulting PPI network was visualized using Cytoscape software. After removing unconnected genes, a PPI network was ultimately constructed, consisting of 21 nodes representing genes and 36 edges representing protein interaction relationships (Figure 3A). Using the MCC, DMNC, MNC, and Degree algorithms in the Cytohubba plugin, 10 key PRGs were screened by each algorithm. By taking the intersection of the results from the four algorithms, seven key PRGs were finally identified (*Fkbp10*, *Tlr2*, *Col1a2*, *Apoe*, *Fstl1*, *Serpinh1*, and *Ppic*) (Figure 3B). In both datasets, the expression of these seven key PRGs was up-regulated in the MI/RI group (Figure 3C,D). The results of the Pearson correlation analysis indicated that the seven key PRGs exhibited strong positive correlations across both datasets (Figure 3E,F).

### 3.4. Identification of Immune Infiltration Landscape in MI/RI

**Figure 4:**
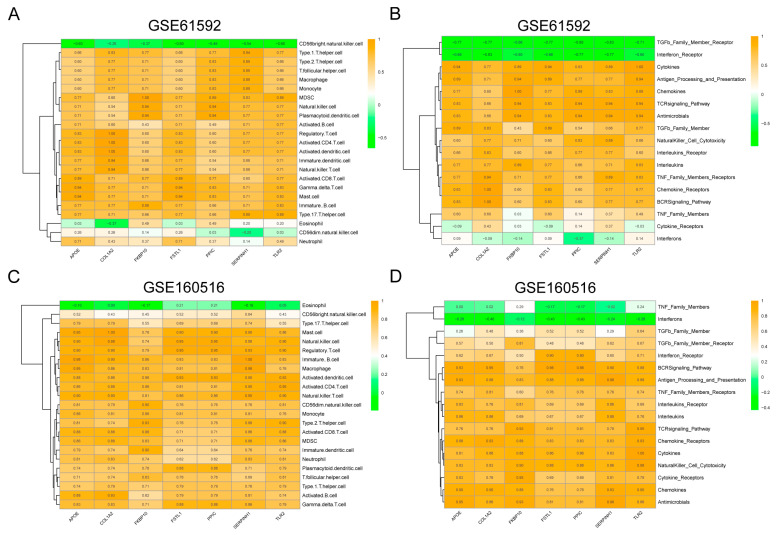
Immune infiltration of key PRGs in MI/RI. (**A**) Immune cell infiltration of key PRGs in GSE61592. (**B**) Immune response infiltration of key PRGs in GSE61592. (**C**) Immune cell infiltration of key PRGs in GSE160516. (**D**) Immune response infiltration of key PRGs in SE160516.

Immune infiltration analysis revealed that in the GSE61592 dataset, the seven key PRGs were negatively correlated with “CD56 bright natural killer cells” and positively correlated with the majority of immune cells, such as “MDSC”, “Natural killer cells”, and “Regulatory T cells” (Figure 4A). In terms of immune responses, the seven key PRGs were negatively correlated with immune responses such as “TGFb Family Member Receptor” and “Interferon Receptor”, while they showed a positive correlation with the majority of immune responses, including “Cytokines” and “Antigen Processing and Presentation” (Figure 4B). In the GSE160516 dataset, the seven key PRGs demonstrated a strong positive correlation with the majority of immune cells, such as “Mast cell”, “Regulatory T cell”, and “Natural killer cell”, while showing a weaker correlation with “Eosinophil” and “CD56bright natural killer cell” (Figure 4C). In terms of immune responses, the seven key PRGs showed a negative correlation with “Interferons” and a strong positive correlation with most immune responses, such as “Antigen Processing and Presentation” and “Cytokines” (Figure 4D).

### 3.5. Molecular Docking of Small Molecular Compounds Targeting Key Genes

**Figure 5:**
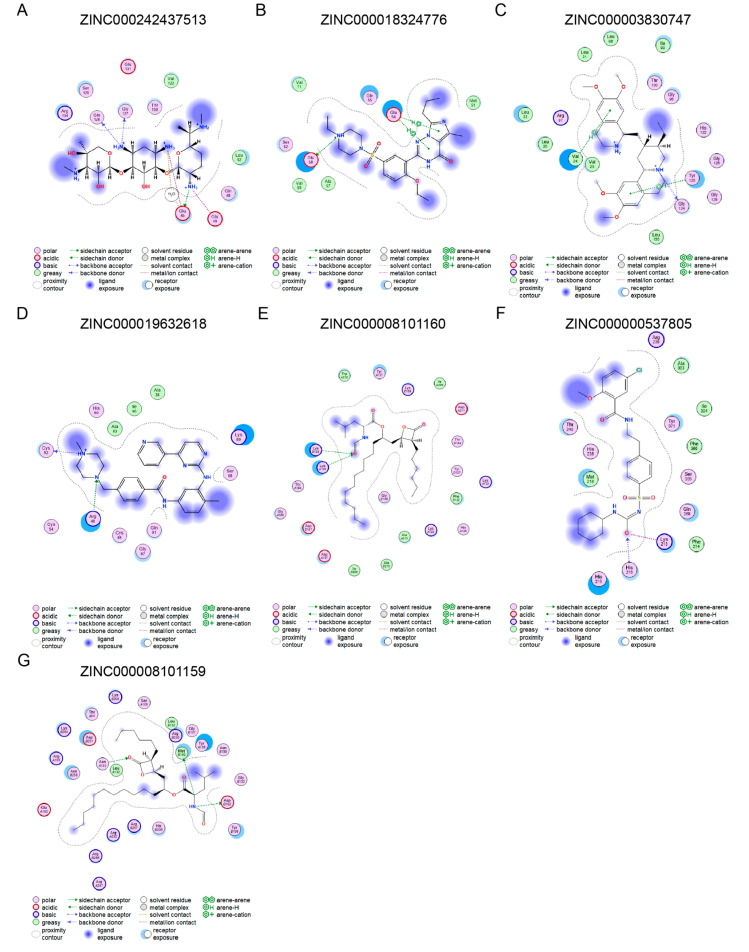
The molecular docking of small molecular compounds targeting key genes. (**A**) ZINC000242437513 is the small molecular compound with the highest docking potential for APOE. (**B**) ZINC000018324776 is the small molecular compound with the highest docking potential for COL1A2. (**C**) ZINC000003830747 is the small molecular compound with the highest docking potential for FKBP10. (**D**) ZINC000019632618 is the small molecular compound with the highest docking potential for FSTL1. (**E**) ZINC000008101160 is the small molecular compound with the highest docking potential for PPIC. (**F**) ZINC000000537805 is the small molecular compound with the highest docking potential for SERPINH1. (**G**) ZINC000008101159 is the small molecular compound with the highest docking potential for TLR2.

The small molecules with the highest docking potential for the seven key genes were screened using MOE software in combination with the ZINC small molecule database. To facilitate better clinical translation, the human 3D molecular structures of the seven key genes were used for docking. The small molecule compound with the highest docking potential for APOE (PDB ID: 1GS9) is ZINC000242437513 (S = −6.0889), which forms a strong hydrogen bond with the active site residue Glu 45 (E = −8.3 kcal/mol) (Figure 5A). The small molecule compound with the highest docking potential for COL1A2 (PDB ID: 5CTD) is ZINC000018324776 (S = −6.1792), which forms a strong hydrogen bond with the active site residue Glu58 (E = −7.3 kcal/mol) (Figure 5B). The small molecule compound corresponding to FKBP10 (AlphaFold predicted) is ZINC000003830747 (S = −8.2048), which forms a hydrogen bond with the active site residue Gly124 (E = −7.3 kcal/mol) (Figure 5C). The small molecular compound predicted to target FSTL1 (AlphaFold predicted) is ZINC000019632618 (S = −6.3969), with a hydrogen bond binding energy of E = −10.9 kcal/mol to the active site Cys52 (Figure 5D). The small molecular compound with the highest docking potential for PPIC (PDB ID = 2ESL) is ZINC000008101160 (S = −8.9526), with a hydrogen bond binding energy of E = −7.7 kcal/mol to the active site Lys125 (Figure 5E). The small molecular compound with the highest docking potential for SERPINH1 (AlphaFold predicted) is ZINC000000537805 (S = −6.9881), with a hydrogen bond binding energy of E = −5.5 kcal/mol to the active site His216 (Figure 5F). The small molecular compound with the highest docking potential for TLR2 (PDB ID = 2Z80) is ZINC000008101159 (S = −9.0594), with a hydrogen bond binding energy of E = −8.4 kcal/mol to the active site Asp182 (Figure 5G). The interactions between these small molecular compounds and the proteins are presented in 2D models. Detailed information on molecular docking can be found in Supplementary File S4.

### 3.6. Validation of Differentially Expressed PRGs in External Dataset

**Figure 6:**
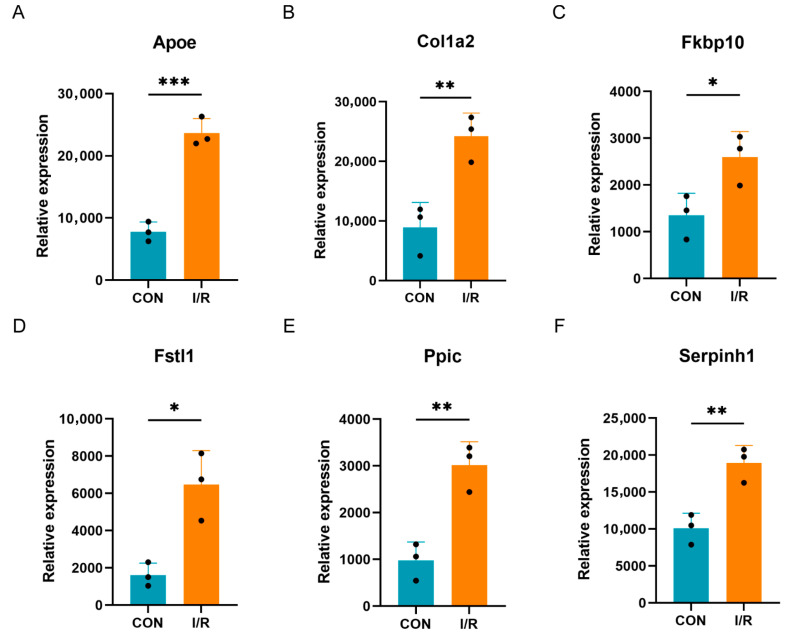
Validation of key PRGs in external datasets. (**A**) Bar plot of mRNA expression of *Apoe* in GSE4105. (**B**) Bar plot of mRNA expression of *Col1a2* in GSE4105. (**C**) Bar plot of mRNA expression of *Fkbp10* in GSE4105. (**D**) Bar plot of mRNA expression of *Fstl1* in GSE4105. (**E**) Bar plot of mRNA expression of *Ppic* in GSE4105. (**F**) Bar plot of mRNA expression of *Serpinh1* in GSE4105. * *p* < 0.05; ** *p* < 0.01; *** *p* < 0.001.

The validation of the expression trends in the seven key PRGs was conducted using the GSE4105 dataset. Excluding *Tlr2*, which was not included in the GSE4105 dataset, the remaining six genes (*Fkbp10*, *Apoe*, *Col1a2*, *Ppic*, *Fstl1*, *Serpinh1*) were all up-regulated in the MI/RI group (Figure 6A–E). The expression trends in these genes in GSE4105 were consistent with those observed in the analysis datasets GSE61592 and GSE160516.

### 3.7. qRT-PCR Validation of Key PRGs in Mouse I/R Model

**Figure 7:**
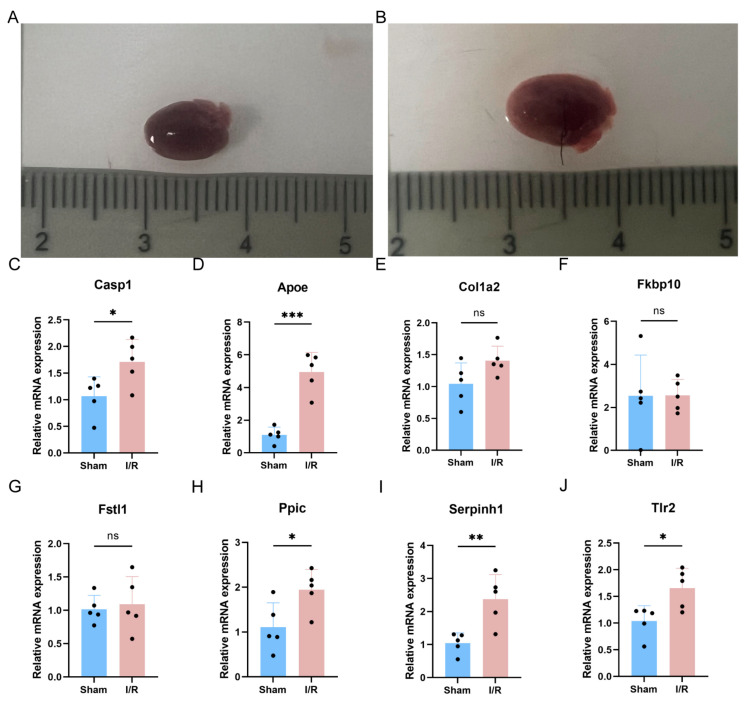
Validation of key PRGs in mouse MI/RI model. (**A**) Photographs of hearts of sham mice. (**B**) Photographs of hearts of MI/RI mice. (**C**) Bar plot of mRNA expression of *Casp1*. (**D**–**J**) Bar plot of mRNA expression of key PRGs. ns *p* > 0.05; * *p* < 0.05; ** *p* < 0.01; *** *p* < 0.001.

To further validate the expression of key PRGs, a sham group and an MI/RI group of mice were constructed (Figure 7A,B). In the mouse model, the expression of the key gene *Casp1*, which is involved in the pyroptosis canonical pathway, was up-regulated (Figure 7C). Additionally, among the seven key genes, *Apoe*, *Ppic*, *Serpinh1*, and *Tlr2* were up-regulated in the mouse model, while *Col1a2*, *Fkbp10*, and *Fstl1* did not show significant differences (Figure 7D–J).

### 3.8. qRT-PCR Validation of Key PRGs in Mouse HL-1 Cell H/R Model

**Figure 8:**
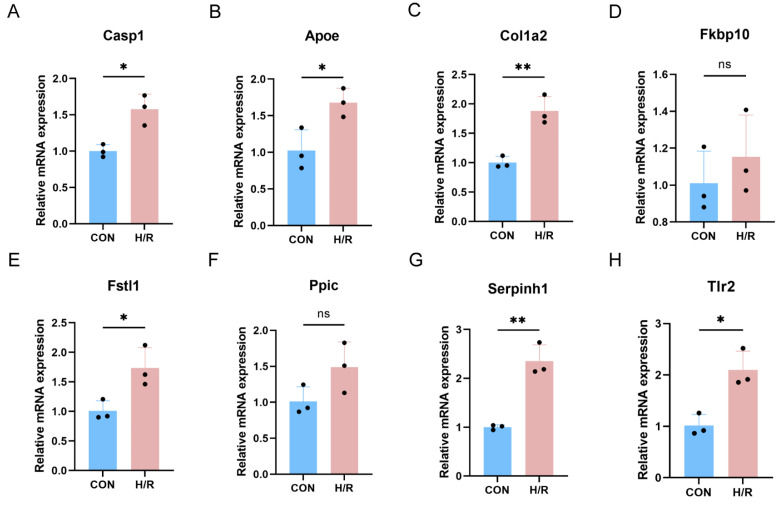
Validation of key PRGs in HL-1 cell H/R model. (**A**) Bar plot of mRNA expression of *Casp1*. (**B**–**H**) Bar plot of mRNA expression of key PRGs. ns *p* > 0.05;* *p* < 0.05; ** *p* < 0.01.

A further validation of key PRGs was performed in the HL-1 cell H/R model (Figure 8A–H). In the cell model, *Casp1* was also up-regulated. Among the seven key PRGs, *Apoe*, *Col1a2*, *Fstl1*, *Serpinh1*, and *Tlr2* were up-regulated in the cell H/R model, while *Fkbp10* and *Ppic* showed no significant difference in expression.

## 4. Discussion

MI/RI is a pathological process in which cardiac tissue damage progressively worsens after coronary artery obstruction is relieved and blood flow is restored. It is also a key factor affecting the prognosis of myocardial infarction patients and adverse cardiac remodeling following reperfusion. In the pathological mechanism of MI/RI, cardiomyocyte death is the fundamental factor that triggers irreversible cardiac damage. In addition, chronic inflammation following MI/RI is also a crucial factor influencing disease progression [19]. Pyroptosis can trigger cell death under pathological conditions while releasing inflammatory factors to promote the inflammatory response. Therefore, targeting pyroptosis in MI/RI may represent a novel approach to simultaneously addressing both cell death and inflammation in the condition.

This study employed bioinformatic methods to mine two MI/RI-related datasets. Differential analysis identified 529 differentially expressed key genes in both datasets. Enrichment analysis indicated that the DEGs primarily participate in biological processes related to “immune system process”, “innate immune system”, and “apoptosis”. The subsequent GO enrichment analysis of 29 PRGs also revealed significant enrichment in multiple immune-related pathways, including “immune system process”, “response to external stimulus”, and “defense response”. These results suggest that the immune system is activated during MI/RI, indicating a strong inflammatory response. Myocardial ischemia induces the intracellular accumulation of sodium, hydrogen, and calcium [20], leading to cellular acidosis and subsequently causing mitochondrial structural damage. The restoration of blood flow leads to the additional generation of reactive oxygen species (ROS) through reoxygenation, further exacerbating tissue damage. Damaged cardiac parenchymal cells and the substances they release are known as damage-associated molecular patterns (DAMPs). These interact with pattern recognition receptors (PRRs) and subsequently activate the innate immune system, leading to inflammatory responses [21]. The NLRP3 inflammasome is a type of PRR. It mediates pyroptosis through the activation of Caspase-1, which then cleaves pro-IL-1β and pro-IL-18 into their mature forms, IL-1β and IL-18, respectively, thus triggering an inflammatory response [22]. Moreover, there is a link between pyroptosis and apoptosis, as Caspase-3 can induce both pyroptosis and apoptosis through GSDME [23]. Furthermore, research indicates that inhibiting the key pyroptosis gene GSDMD can enhance the activation of the DNA repair enzyme PARP1, which subsequently promotes apoptosis [24]. Current studies report that MI/RI primarily induces pyroptosis through the NLRP3/CASP1/GSDMD-mediated canonical pathway [25,26,27], whereas non-canonical pyroptosis has not been documented in MI/RI.

Inflammation plays a critical role in the onset and progression of cardiovascular diseases (CVDs). It is not only a contributing factor to the development of CVD but also an important determinant of its prognosis. Clinically, several classical inflammatory biomarkers, such as C-reactive protein, IL6, and TNFα, have been widely used as diagnostic and prognostic indicators of CVD. With the advancement of research in the field of inflammation, a number of novel biomarkers—such as sirtuins, miRNAs, ST2, ApoE, and adiponectin—have also been proposed for use in the diagnosis and prognostic evaluation of cardiovascular diseases [28]. Inflammation can also interfere with or exacerbate other diseases. Research has demonstrated that the excessive activation of inflammation, along with adverse cardiovascular outcomes, contributes to abdominal fat accumulation in diabetic patients [29]. Moreover, inflammatory activation during MI/RI can also contribute to the progression of other diseases, such as coronary microvascular dysfunction in diabetes, thereby exacerbating cardiac injury [30]. Similarly, inflammation also plays a critical role in gender-related cardiovascular diseases. In premenopausal women, both fatty and non-fatty breasts exhibit the overexpression of SGLT2 and inflammatory cytokines, along with the down-regulation of breast sirtuins, which significantly contributes to adverse cardiovascular outcomes [31].

We identified 29 differentially expressed PRGs from the key genes, and enrichment analysis indicated that these genes are associated with processes such as “immune system process” and “response to stress”. This suggests that PRGs in MI/RI are closely related to immune processes. Subsequently, we further identified seven key PRGs (*Fkbp10*, *Tlr2*, *Col1a2*, *Apoe*, *Fstl1*, *Serpinh1*, and *Ppic*) through PPI analysis and conducted immune infiltration analysis to explore their relationships with immune cells and immune responses. Through animal experiments and cellular studies, we conclusively demonstrated that the expression patterns of *Apoe*, *Tlr2*, and *Serpinh1* in both mouse cardiac I/R injury models and cardiomyocyte H/R models were consistent with our analytical predictions. *Apoe* is an apolipoprotein that is a component of chylomicrons, very-low-density lipoproteins (VLDLs), and high-density lipoproteins (HDLs). It is closely associated with Alzheimer’s disease and ischemic heart disease [32]. A study has shown that the activation of the LXR/ApoE signaling pathway in tumors can inhibit the survival of myeloid-derived suppressor cells (MDSCs) [33]. *Col1a2* is a major component of type I collagen. Studies have shown that *Col1a2* is positively correlated with immune infiltration in colon adenocarcinoma [34] and is considered a key immune-related gene in dilated cardiomyopathy [35]. *Tlr2* is well-known for its crucial role in activating the innate immune response. Research has demonstrated that MI/RI is mediated by *Tlr2*, and anti-*Tlr2* therapy can significantly reduce immune cell infiltration in MI/RI [36]. These studies highlight the regulatory potential of these genes in the immune response during MI/RI, suggesting that further research is necessary to explore their precise mechanisms.

Subsequently, we validated the seven key PRGs and *Casp1*, which is among the differentially expressed PRGs, using qPCR in a mouse MI/RI model and a mouse HL-1 cell H/R model. In both the animal model and cell model, *Casp1* was up-regulated in the experimental group, suggesting that pyroptosis mediated by MI/RI may be triggered by the *Casp1*/NLRP3/GSDMD axis canonical pathway. A study found that silencing the NLRP3/*Casp1* axis through calpain can alleviate MI/RI, which further supports our research hypothesis [37].

In the MI/RI mouse model, the expression of *Apoe*, *Tlr2*, *Col1a2*, *Serpinh1*, and *Ppic* was up-regulated in the experimental group. In the HL-1 cell H/R model, the expression of *Apoe*, *Col1a2*, *Tlr2*, *Serpinh1*, and *Fstl1* was up-regulated in the experimental group. It can be demonstrated that *Apoe*, *Tlr2*, and *Serpinh1* exhibit a consistent up-regulation trend in MI/RI. *Apoe* deficiency leads to impaired lipoprotein clearance, making *Apoe*-deficient mice commonly used in constructing atherosclerosis models [38]. Studies have demonstrated the presence of GSDMD-mediated aortic endothelial cell pyroptosis [39] and GSDME-mediated macrophage pyroptosis in *Apoe*-deficient atherosclerotic mice [40]. Meanwhile, studies also show that *Apoe* deficiency promotes lipid deposition and neutrophil overactivation, increasing inflammatory cytokine release and aggravating post-MI/RI inflammation [41,42]. In allergic airway inflammation, *Tlr2*-deficient rats exhibit reduced pyroptosis. Additionally, in doxorubicin-induced cardiac injury, the up-regulation of *Tlr2* can stimulate pyroptosis in cardiomyocytes [43]. Moreover, TLR2 has been widely recognized to mediate pyroptosis. Upon the recognition of bacterial lipopolysaccharides (LPSs), it activates the NF-κB pathway, thereby inducing NLRP3 inflammasome-mediated canonical pyroptosis [44]. *Serpinh1* encodes a member of the serine protease inhibitor family that primarily functions as a collagen-specific molecular chaperone during collagen biosynthesis. In an osteosarcoma study, *Serpinh1* was demonstrated to exhibit positive correlations with multiple programmed cell death pathways, including pyroptosis, apoptosis, and ferroptosis [45]. In another study, the opioid peptide SLP was shown to suppress the expression of *Serpinh1* following myocardial ischemia/reperfusion injury (MI/RI) in mice [46]. The above study revealed the role of key PRGs in the pathogenesis of pyrogen in different diseases, but whether they participate in the pyrogen process of MI/RI by similar mechanisms still needs further investigation.

This study, based on transcriptome data mining, external dataset validation, and verification through animal and cell experiments, still has certain limitations. The key genes we identified have not been validated for their phenotypic effects through knockdown or overexpression experiments, and the molecular docking results have not been experimentally confirmed in subsequent studies. The immune infiltration analysis based on ssGSEA is solely derived from bulk RNA-seq data and does not take into account the immune phenotypic differences resulting from distinct cell types. In our animal experiments, inflammation is the result of interactions among multiple cell types, whereas in the cell experiments, inflammatory cytokines are produced by cardiomyocytes through autocrine or paracrine mechanisms. Therefore, there are inherent differences between the two models. Moreover, since cell-based experiments cannot fully replicate the complex microenvironment present in animal models, certain genes exhibited inconsistent expression trends between in vivo and in vitro systems. In the animal experiments, we only established the animal model without measuring the infarct size. The conclusions drawn in this study are exploratory and require further experimental validation in subsequent research. In future studies, we will establish transgenic animal models to validate the effects of each key gene on the pyroptosis phenotype and perform drug-based experiments to verify the results of the molecular docking analysis.

## 5. Conclusions

This study employed bioinformatic methods to screen PRGs within MI/RI-related datasets, exploring their associated signaling pathways, immune infiltration, and key genes. Docking models of potential small molecular compounds targeting the key genes were constructed. Validation was conducted using external datasets, animal models, and cell models. Ultimately, *Apoe*, *Serpinh1*, and *Tlr2* were identified as key PRGs in MI/RI. These identified genes may provide new research directions for investigating the pyroptosis pathway in MI/RI.

## Data Availability

The datasets presented in this study can be found in the Gene Expression Omnibus (GEO) database (https://www.ncbi.nlm.nih.gov/geo). The accession number(s) can be found in the article.

## Supplementary Materials

The following supporting information can be downloaded at https://www.mdpi.com/article/10.3390/biomedicines13092114/s1. File S1: The details of all primers. File S2: Detailed information about the key genes. File S3: The detailed information about the differential expression PRGs. File S4: The detailed information on molecular docking. The datasets presented in this study can be found in online repositories. The names of the repository/repositories and accession number(s) can be found in the article/Supplementary Material.

## Abbreviations

The following abbreviations are used in this manuscript:

BP	Biological Process
CC	Cellular Component
DEG	Differentially Expressed Gene
GEO	Gene Expression Omnibus
GO	Gene Ontology
H/R	Hypoxia–Reoxygenation
KEGG	Kyoto Encyclopedia of Genes and Genomes
MF	Molecular Function
MI/RI	Myocardial Ischemia–Reperfusion Injury
MOE	Molecular Operating Environment
PPI	Protein–Protein Interaction
PRG	Pyroptosis-Related Gene
